# Bacterial Amyloids as Hubs for Nucleic Acid Interactions: Implications and Mechanisms

**DOI:** 10.3390/ijms26146560

**Published:** 2025-07-08

**Authors:** Sylwia Bloch, Gaelle Loutfi, Gautier Moroy, Richard R. Sinden, Grzegorz Węgrzyn, Véronique Arluison

**Affiliations:** 1 Department of Molecular Biology, University of Gdansk, Wita Stwosza 59, 80-308 Gdansk, Poland; sylwia.bloch@ug.edu.pl; 2 Laboratoire Léon Brillouin, UMR 12 CEA/CNRS, Bâtiment 563, Site de Saclay, 91191 Gif-sur-Yvette, France; gaelle.loutfi@etu.u-paris.fr; 3 Université Paris Cité, CNRS, INSERM, Unité de Biologie Fonctionnelle et Adaptative, 75013 Paris, France; gautier.moroy@u-paris.fr; 4 Department of Chemistry, Biology and Health Sciences, South Dakota School of Mines and Technology, Rapid City, SD 57701, USA; rrsinden@gmail.com; 5 Université Paris Cité, UFR SDV, 35 Rue Hélène Brion, 75013 Paris, France

**Keywords:** bacterial amyloids, DNA and RNA transactions, nucleoid, small noncoding RNA (sRNA), curli, extracellular DNA (eDNA), biofilm, liquid–liquid phase separation (LLPS), Hfq, RepA, TmaR, CsgA

## Abstract

Amyloids are protein aggregates having a cross-β structure, and they reveal some unusual properties, like interactions with specific dyes and resistance to actions of detergents and proteases, as well as the capability to force some proteins to change their conformation from a soluble form to aggregates. The occurrence of amyloids is not restricted to humans and animals, as they also exist in microbial cells. However, contrary to animals, where amyloids are usually pathological molecules, bacterial amyloids are often functional, participating in various physiological processes. In this review, we focus on a specific property of bacterial amyloids, namely their ability to interact with nucleic acids and resultant regulatory mechanisms. Moreover, some of these interactions might play indirect roles in the pathomechanisms of human neurodegenerative and inflammatory diseases; these aspects are also summarized and discussed in this review.

## 1. Introduction

Amyloids are typically defined as a class of protein aggregates that are insoluble in water, exhibit an ordered structure, and form non-crystalline self-assemblies. These aggregates are generally characterized by a cross-β structure [1,2,3]. However, some properties of these proteins are non-canonical. These include high resistance to detergents and proteases [4,5], the ability to interact with specific dyes [6], and characteristics that promote the transition of some proteins from a soluble to an aggregated form [7,8]. On the other hand, similar properties have also been observed in protein aggregates that do not contain a cross-β structure in their structures. These proteins are usually referred to as “amyloid-like” [2]. According to the International Society of Amyloidosis nomenclature, amyloids are primarily defined by the presence of a cross-β structure, while the presence of the other above-mentioned properties allows the assignment of proteins as amyloid-like aggregates only [9]. The example of cross-α amyloid structures illustrates the amyloid-like family [10,11].

Although amyloids are commonly associated with neurodegenerative diseases, such as Alzheimer’s, associated with amyloid-β peptide [12], research over the past two decades has shown that similar structures can also occur in bacterial cells [13]. Contrary to amyloids found in humans and animals, which are usually recognized as pathogenic agents, bacterial amyloids can be functional bio-assemblies [14], participating in various physiological processes [15]. These include regulation of DNA replication and the cell-cycle, contribution to structural scaffolds, participation in cell adhesion processes, and modulation of host–pathogen interactions [13,15]. Some of these functions are mediated by an interesting feature of microbial amyloids, namely interactions with nucleic acids, a property common with pathological amyloids [16]. In this review, we focus on these properties, discussing their contribution to the regulation of bacterial physiology. In addition, we discuss the potential roles of bacterial amyloids in the development of some human diseases, including neurodegenerative and inflammatory disorders.

## 2. Methods Used in This Work

This article is a kind of a narrative review. It is based on published data, included in publications presented in the English language, which were recorded in the PubMed database (https://pubmed.ncbi.nlm.nih.gov/; last accessed on 7 June 2025). The term “bacterial amyloids and nucleic acids” was used for the literature search. In this search, the number of records found was 214. Publications that did not address the problem of interactions of bacterial amyloids with nucleic acids, as well as articles published in languages other than English, were excluded. As a result of such a selection, 82 articles were subjected to detailed analysis. In this paper, some other articles are also cited that concern more general aspects of amyloid.

## 3. Bacterial Amyloid–Nucleic Acid Interactions: Mechanisms and Roles in Microbial Cells

About 25 years ago, a team of researchers led by Akira Ishihama investigated the intracellular distributions of DNA-binding proteins in *Escherichia coli* using immuno-fluorescence methods. They identified two distinct groups of such proteins: one group consisted of polypeptides uniformly distributed along the bacterial chromosome, while the other exhibited an irregular distribution, forming spots or clumps [17]. At the time, they did not consider the possibility that some of these foci might represent amyloidal structures interacting with bacterial DNA. However, we suspect that at least some of these foci could actually be bacterial amyloids interacting with nucleic acids in bacterial cells [18,19,20,21,22,23]. A transformative discovery in cell biology has revealed that proteins and RNA and DNA can self-organize into membrane-less compartments known as biomolecular condensates through liquid–liquid phase separation (LLPS) [24]. These dynamic structures help cells spatially organize their internal components and locally concentrate macromolecules to optimize biochemical processes [25,26]. Recent studies have revealed that amyloidogenic proteins can undergo LLPS, suggesting that amyloid formation may play roles in normal physiological processes [27]. Today, we know that proteins with amyloid-like structures can interact with the bacterial nucleoid and influence its topology. This is exemplified by the DNA-compacting effects of the *E. coli* Hfq protein [28] (see also below). Nevertheless, we suggest that the work by Ishihama et al. provided the first evidence—albeit unrecognized at the time—that amyloids in bacterial cells might interact with nucleic acids. Simultaneously, in the same year, the physiological function of amyloids was demonstrated, leading to the introduction of the term “functional amyloid” [14,29]. However, that work was based on experiments conducted with yeast cells. The first report on bacterial functional amyloids came two years later when the group of Scott J. Hultgren demonstrated that the extracellular matrix that holds Gram-negative cells into a persistent biofilm is composed of protein and possibly DNA [30]. This matrix includes curli with a cross-β architecture [31]. Subsequent studies, some of which are discussed below, have confirmed that amyloids can play important roles in bacterial physiology [32].

Extrachromosomal genetic elements in bacterial cells, such as plasmids and bacteriophage genomes, have played fundamental roles in numerous groundbreaking discoveries in biochemistry, genetics, and molecular biology. A similar story unfolded in the discovery of bacterial amyloids interacting with nucleic acids. In studies on the RepA protein, which functions as an initiator of DNA replication for plasmid pPS10, specific nanostructures such as irregular aggregates, fibers, and amyloid spheroids formed upon interaction with double-stranded DNA oligonucleotides [33]. Rafael Giraldo was a pioneer in this field, and his research team later demonstrated that the winged-helix dimerization (WH1) domains of RepA are responsible for amyloid formation, and that DNA can promote the assembly of RepA into amyloids [34]. Although RepA amyloid structures appeared to play important roles in controlling pPS10 DNA replication, when a hyper-amyloidogenic functional variant of RepA was constructed, an amyloid proteinopathy was observed in *E. coli* cells [35]. These results could be interpreted as evidence that, despite the functional importance of RepA amyloids, an excess of these amyloid structures may be toxic to bacterial cells—somewhat similar to the accumulation of amyloid proteins, such as β-amyloid in Alzheimer’s disease, in human organisms. Indeed, bacterial proteins can misfold and form amyloid-like aggregates that can influence cell death through several mechanisms, including programmed cell death (PCD) [36]. The physiological relevance of PCD is disputable, with two predominant hypotheses defined as ‘cellular altruism’ (to eliminate damaged cells from the population, thus enhancing the survival rate of the rest of the bacterial population due to conferment of fitness to kin), and ‘genetic selfism’ (death due to loss of functions of selfish genes) [37]. Nevertheless, PCD was proposed to be a potential basis for developing new antibiotics [38]. Indeed, one might easily imagine that the formation of deleterious amyloids inside pathogenic bacterial cells might lead to their death. The problem is to find an efficient way to stimulate the formation of hyper-amyloidogenic structures inside such cells. Another example of bacterial cell death caused by amyloidal structures is a deleterious effect of proteins containing a C-terminal amyloid domain involved in signal transmission, so called NLR signalosomes (nucleotide binding and leucine-rich repeat-containing receptors); these can be involved in cell death execution by forming pores in cellular membranes [39]. Therefore, it is also plausible that specific anti-bacterial drugs might be developed employing such a mechanism of action of proteins bearing specific amyloid domains.

Another similarity between RepA WH1-domain amyloids and human β-amyloid was discovered during structural studies. The WH1 domain mimics the hierarchical assembly of human amyloidogenic proteins in the formation of filaments at the origin of plasmid DNA replication [40]. Importantly, the role of nucleic acid was suggested to be crucial, as the nucleoid was identified as the site where oligomeric amyloid precursors of RepA-WH1 are generated [41]. In fact, the regulatory role of RepA WH1 amyloids is negative, as these structures are responsible for the “handcuffing” mechanism that inhibits plasmid replication initiation, thus preventing uncontrolled increases in plasmid DNA copy number, which could be toxic to bacterial cells [42].

While the WH1 domain of the RepA protein encoded by plasmid pPS10 was the first bacterial amyloid to function in connection with DNA transactions, subsequent studies have demonstrated that such amyloid–nucleic acid functional interactions may be widespread in the prokaryotic world. Another example comes from studies on the extracellular matrices of biofilms, which are structures that allow bacteria to form multicellular aggregates and protect against various physical, chemical, and biological agents [43,44]. Note that amyloid formation is generally considered to be an irreversible process. However, recent findings suggest that the initial step of amyloidogenesis, characterized by the formation of oligomers (~10–50 monomers), could be reversible, whereas the formation of larger aggregates is not [45]. This reversibility might also apply to bacterial amyloids. Furthermore, bacteria may possess mechanisms to disassemble amyloid fibers. For instance, in biofilms, some bacteria produce enzymes or molecules that can disrupt the biofilm matrix and potentially reverse amyloid formation [46]. Active research is thus being conducted to develop therapeutic strategies to target and disrupt bacterial amyloids. Nevertheless, achieving true reversibility of amyloid fibrils remains a significant challenge. Among other components, extracellular DNA (eDNA) is a structural element of biofilms. Interestingly, in the presence of DNA, the beta toxin of *Staphylococcus aureus* forms covalent self-cross-links [47], suggesting that these structures may resemble amyloids. Further studies demonstrated that the formation of amyloid fibers is promoted in the presence of eDNA in biofilms produced by *S. aureus*, although other proteins, rather than beta toxin, were implicated in this phenomenon [48]. It should be noted that eDNA-bound amyloid structures are important components of biofilms formed by other bacteria as well, contributing specific characteristics to these multi-component complexes and playing crucial defensive roles in the physiology of different bacterial species [49,50]. Note that amyloid-bound eDNA may form non-canonical DNA structures like Z-DNA or G-quadruplexes [51,52]. Curli, which are proteinaceous components of biofilms, can form amyloid structures [30]. Importantly, the presence of eDNA resulted in increased sizes of curli intermediates [53], suggesting direct interactions between these nucleic acids and amyloidogenic curli. When curli are produced by pathogenic bacteria, their complexes with DNA may act as virulence factors, with curli amyloids behaving as nucleic acid chaperones [54,55,56].

Bacterial amyloids can interact not only with DNA but also with RNA. The PrD domain of the Rho factor, an RNA-binding protein involved in regulating transcription termination [57], can self-assemble into amyloid-like structures in *Clostridium botulinum* [58,59]. This might significantly impact the regulation of gene expression efficiency in this bacterium, particularly under various stress conditions. Other gene expression regulators can also form amyloids. An interesting example is the CarD protein of *Mycobacterium tuberculosis*, an essential global transcription regulator that stabilizes the transcription initiation complex. CarD has a tendency to form amyloid-like fibrils, as confirmed in both in vitro and in vivo experiments [60]. A similar formation of amyloid-like fibrils was observed with the HelD protein of *Bacillus subtilis* [61]. HelD is a helicase that interacts with RNA polymerase and stimulates transcription. The LARA domain of the *E. coli* RavA protein, a chaperone-like ATPase, can also form amyloids, particularly under conditions of low pH or elevated temperature [62]. This amyloid formation could influence gene expression by modulating the chaperone activity of the protein.

Hfq is a bacterial protein originally discovered as an RNA chaperone but later identified as a multifunctional protein involved in various biological activities [63]. This small protein (102 amino acid residues in *E. coli*) consists of two domains, N-terminal and C-terminal, with the latter forming an amyloid structure [64,65,66]. The C-terminal region of Hfq is intrinsically disordered but prone to aggregation under certain conditions [67]. Experiments conducted over the past decade have shown that Hfq is involved not only in RNA biology but also in DNA replication, particularly in certain replicons, such as plasmids [68]. Hfq can interact directly with DNA, which may have significant consequences for DNA structure, influencing various biological processes. Approximately 20% of cellular Hfq is associated with DNA [69], and Hfq can form fiber-like structures [70]. This filamentous organization is mediated by the C-terminal region of Hfq, which possesses amyloid-like properties that facilitate its assembly on the chromosome, contributing to DNA compaction [71,72,73,74,75]. However, previous observations of altered DNA topology in Hfq deletion strains are now attributed to indirect regulatory effects on other proteins, as Hfq itself does not directly modify DNA topology [71]. While specific details on the distribution of amyloids to daughter cells during bacterial cell division are scarce, nucleoid-bound amyloids, as in the case of Hfq, opens the possibility for a direct transfer of an amyloid structure to progeny. Interestingly, Hfq can also interact with cell membranes. The C-terminal amyloid-like domain of this protein plays a crucial role in maintaining membrane integrity [76], which is important in light of recent discussions about the interconnection of DNA transactions with cell membranes, especially in bacteria [77]. Note that Hfq porates bacterial membranes, and in conjunction with Omp porins they could influence DNA uptake [78]. Indeed, both OmpC and OmpF can adopt an amyloid structure that is alternative to their functional β-barrel form [79], and the CTR of Hfq may influence the equilibrium between the β-barrel and amyloid forms of the porins. The potential physiological mechanism of amyloid DNA delivery indeed remains an open question and warrants further exploration [80].

Hfq interacts not only with double-stranded DNA but also with single-stranded DNA molecules, contributing to the regulation of DNA replication (especially in bacteriophages with single-stranded DNA genomes) and recombination. Moreover, the C-terminal amyloid domain can significantly alter the structure of single-stranded DNA and its helical parameters [81]. Very recent studies revealed another crucial property of the amyloid part of Hfq—it can bridge and compact DNA molecules [75]. These discoveries led to the conclusion that bacterial amyloids might play a role in both genome architecture and the regulation of gene expression efficiency by modifying the shape of the DNA molecule.

Regarding interactions between Hfq and RNA molecules, the protein was initially identified as a host factor essential for Qβ phage infection, where it facilitates the efficient replication of *E. coli* Qβ bacteriophage RNA by disrupting the secondary structure of the Qβ plus-strand RNA [82,83,84]. Today, Hfq is known to function as an RNA hub that facilitates imperfect base-pairing between trans-encoded sRNAs and their mRNA targets [64,85,86,87,88]. The C-terminal domain was long considered dispensable for the interaction with RNA [89,90]. However, more recent studies clearly demonstrate that the C-terminal amyloid region of the *E. coli* Hfq protein significantly influences its binding with RNA, modulating the effects of the protein on RNA annealing [91,92,93,94]. Furthermore, Hfq transiently binds its RNA substrates by cycling them on and off, and its C-terminus may help this cycling [92,95]. In addition to its other myriad activities, Hfq is also involved in interactions with ribosomes and rRNAs [96]. It is critical for ribosome biogenesis by participating in the formation of mature 16S rRNA and 30S ribosomal subunits from precursors [97]. Moreover, Hfq, in conjunction with RNase R, is responsible for processing 16S and 23S precursor rRNAs into functional 16S and 23S rRNAs [98]. These activities are critical for the formation of functional 70S ribosomes. Significantly, a lack of Hfq leads to suboptimal ribosomes, resulting in a decrease in both the efficiency and the fidelity of translation [97].

Additionally, Hfq can interact with the Rho transcription terminator [99] (see above), and a possible cross-seeding between the amyloid structures may occur in vivo [100]. Besides Rho, Hfq has also been reported to associate with many proteins involved in RNA metabolism, such as RNaseE, poly(A)polymerase, and S1 and RNA polymerase [101,102,103]. Intrinsically disordered proteins or regions (IDP/IDR) such as the Hfq C-terminal region indeed utilize a variety of binding mechanisms that mediate interactions with protein and RNA partners, coupling binding and folding where the disordered protein evolves to an ordered conformation, such as an amyloid, upon binding [104]. This may occur with the Hfq C-terminus. In summary, the C-terminal region of the Hfq protein interacts with both DNA and RNA, including double-stranded and single-stranded regions of nucleic acids. These interactions significantly affect the topology of these nucleic acids, greatly influencing crucial DNA- and RNA-specific processes such as DNA replication, genetic recombination, and gene expression efficiency, especially at the stages of transcription and RNA stability control.

Recently, Orna Amster-Choder’s group identified TmaR, a sugar metabolism regulator, as the pole-localizing factor in *E. coli* [105]. They subsequently discovered that TmaR undergoes LLPS via interactions with RNA. Through this mechanism, TmaR extends its regulatory functions beyond sugar uptake, influencing the expression of flagellar proteins and thus affecting bacterial motility and biofilm development [106,107]. Interestingly, their recent studies demonstrate that TmaR could, under certain conditions, form filamentous structures suspected to be amyloid [107].

Finally, an intriguing article was published in 2023, where peptide amyloids (in the length of several amino acid residues) were demonstrated to bind RNA molecules with a length as short as three nucleotides. Moreover, such interactions were sequence-dependent, suggesting a high specificity [108]. Therefore, one might suggest that amyloid-RNA interactions could be considered as a process playing crucial roles in the development of life, at its origin stage. Since the above-described interactions were sequence-specific, it is possible that the appearance of the genetic code was influenced by this kind of intramolecular transactions.

## 4. Roles of Bacterial Amyloids and Their Interactions with Nucleic Acids in the Development of Neurodegenerative Diseases

Our current understanding of the causes of neurodegenerative diseases involves the role of amyloids as compounds toxic to nerve cells. In particular, β-amyloid is considered one of the main molecules responsible for the development of Alzheimer’s disease [109]. It has been proposed that amyloids occurring in the human body could directly interact with amyloids of bacterial origin [110]. Such interactions might enhance the aggregation efficiency and facilitate the more effective accumulation of amyloid fibrils [109]. Nevertheless, the DNA component also appears to play an important role in amyloid formation. Specifically, fragments of DNA derived from spirochetes, a specific class of bacteria, have been found alongside amyloids in both biofilms of spirochetal origin and in Alzheimer’s disease senile plaques [111]. Therefore, one might speculate that the interactions between amyloids and DNA play crucial roles in the formation of these structures, suggesting that the contribution of spirochetal amyloids and their binding to nucleic acids could be a potential cause of neurodegenerative disease(s). Intriguingly, it has been reported that extracellular DNA of bacterial origin can trigger β-amyloid aggregation [112]. Moreover, similar effects of bacterial DNA on the appearance of tau-protein aggregates have been observed. Surprisingly, the source of extracellular DNA appears to be crucial, as nucleic acid fragments derived from certain bacterial species (but not all) produced the effects described above [112].

It has been proposed that interactions between bacterial functional amyloids derived from bacteria occurring in the human microbiome and human pathological amylodis contribute significantly to spreading the latter class of molecules. Namely, such interactions might stimulate the transmission of human pathological amyloids from cell to cell through small fragments of amyloid molecules, triggering the growth of new amyloids when encountering monomeric amyloid precursors [110]. In fact, bacterial amyloids reveal a similarity to β-amyloid, as both types of molecule are more cytotoxic to macrophages in the form of oligomers than the mature aggregates [53]. Curli, produced mostly by bacteria-forming biofilms, can be recognized as amyloid-like proteins, and they show not only structural [113] but also functional similarities to human β-amyloid. Using a murine model, evidence of vagus nerve activation was demonstrated in response to bacterial curli [114]. This suggests that gut–vagus–brain signaling may play a role in the development of spreading pathological amylodis in mammals. Importantly, bacterial curli (with their amyloid structures) and human amyloids can be recognized by the same receptors; thus, curli can induce inflammatory processes, including neuroinflammation, which is involved in developing neurodegenerative diseases. Furthermore, curli can participate in the self-assembly of pathological human amyloids [56]. The linking of the gut–brain axis to the development of Alzheimer disease was corroborated in another study with a mouse model, where the contribution of a specific bacterial species, *Faecalibacterium prausnitzii,* was strongly suggested [115].

An interesting proposal was published suggesting that the WH1 amyloid domain of the prokaryotic RepA protein (a replication initiator protein required for pPS10 plasmid DNA replication; described in more detail in Section 3) could be used as a model in studies on human diseases caused by the accumulation of amyloids [116]. Apart from interacting with DNA, which might trigger amyloid fiber formation through DNA–protein interactions, the WH1 domain can form co-aggregates with crucial proteins involved in cellular defense mechanisms (e.g., oxidative stress response). This leads to the sequestration of these proteins, reducing the efficiency of stress responses and increasing the likelihood of aggregate accumulation, including amyloids [116].

## 5. Bacterial Amyloids Interacting with Nucleic Acids as Factors Playing Roles in Inflammatory Disorders

As mentioned in the preceding sections, bacterial curli, which are structures acting as crucial components of biofilms, especially when interacting with extracellular DNA, adopt an amyloid form [56]. Apart from constituting the foundation of biofilms, curli–DNA complexes can trigger an immune response and the production of autoantibodies in mice [117]. Thus, one might speculate that autoimmune diseases could be caused, at least in part, by bacterial infections and the formation of biofilms containing curli and extracellular DNA. More detailed studies have indicated that the immune response stimulation by curli and DNA arises from the curli–DNA complex-dependent activation of two receptors: Toll-like receptor 2 (TLR2) at the cell surface and, after internalization, Toll-like receptor 9 (TLR9) [118], as well as the NLRP3 inflammasome [119,120,121]. Pathological inflammatory processes, such as joint inflammation, have been observed in both laboratory animals and humans suffering from infectious diseases in the presence of curli–DNA complexes [55,122,123].

## 6. Methodology of Studies on Interactions Between Bacterial Amyloids and Nucleic Acids

**Figure 1:**
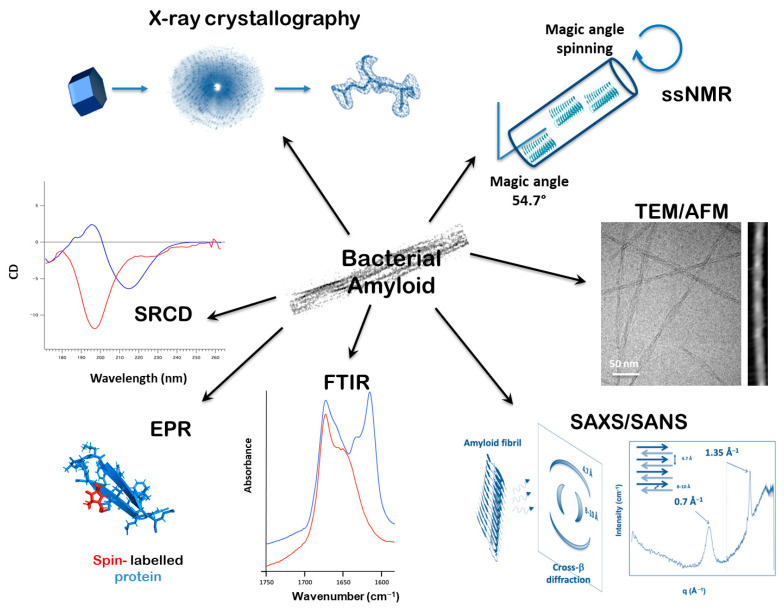
Summary of methodologies used to study bacterial amyloids and their interaction with nucleic acids. These include techniques that provide information of a global (nm) or local (Å) scale. X-ray crystallography and ssNMR analysis of amyloid peptides can provide detailed three-dimensional information regarding structural organization at the atomic level. Molecular imaging using AFM and TEM can reveal the structures of fibers on a flat surface on a nm scale. Recent advances in cryo-EM even allow one to reach the Å-scale. Using small angle X-ray or neutron scattering (SAXS/SANS), the reflections corresponding to cross-β structure can be seen at ~0.7 and ~1.35 Å^−1^, corresponding to inter-sheet and inter-strand distances of 8–10 Å and 4.7 Å. FTIR infrared spectroscopy reveals the structural features of protein secondary structures and nucleic acid conformations by monitoring bond vibrations (red line non amyloid, *vs* blue line amyloid, same for SRCD). Electron paramagnetic resonance (EPR) relies on interactions between unpaired electrons and an external magnetic field, and using paramagnetic tags attached to amyloid fibrils, information about flexibility and rigidity can be ascertained. Spectra from synchrotron radiation circular dichroism (SRCD) can inform aspects of β-sheets secondary structure as well as the specifics of nucleic acid conformation.

A range of in vivo and in vitro techniques are employed to investigate amyloid–nucleic acid interactions. They are summarized graphically in Figure 1. The major challenge is determining high-resolution 3D structures of these complexed amyloid fibrils. Amyloids are notoriously difficult to crystallize for X-ray diffraction, and X-ray crystallography typically relies on the use of short peptides [124], which form β-strands stacked at 4.7–4.8 Å—the hallmark of the amyloid architecture known as the cross-β structure [1] (Figure 1). However, these peptide crystals are often small and fragile, requiring delicate handling. Recently, micro-electron diffraction has, however, emerged as a complementary technique, allowing structural analysis of these submicron crystals [125]. Nevertheless, small peptides frequently lack the NA-binding properties of the full proteins (see, as an example, the 11 amino acid residues that form the basis of amyloidogenicity [126]).

A powerful tool to analyze amyloids is solid-state nuclear magnetic resonance (ssNMR) spectroscopy. Based on the same fundamental principle as solution NMR—the interaction between nuclear spins and a magnetic field—ssNMR provides detailed insights into molecular distances within insoluble systems, such as membrane proteins and amyloid fibrils [127,128,129]. In a solid (here amyloid fiber), where nuclei are in fixed positions, signals broaden significantly. To overcome this, ssNMR employs magic angle spinning, where the sample is spun rapidly at an angle of 54.74° relative to the magnetic field. This averages out anisotropic interactions and sharpens the signal [130]. While ssNMR enables 3D structural elucidation, its application to DNA:amyloid complexes is limited due to the requirement for isotopic labeling, associated with high costs and the need for specific labelled molecules, which are not always available commercially.

Cryo-electron microscopy (cryo-EM) has gained prominence for resolving the structures of amyloids and amyloid:NA complexes at near-atomic resolution [131,132]. Traditional EM remains a fast and effective method to assess amyloid fibril morphology—including length, width, and intertwining. Negative staining, using uranyl acetate or gadolinium salts on carbon-coated grids, provides contrast for rapid imaging at high magnification. In contrast to classic EM, cryo-EM involves flash-freezing fibrils in vitreous ice, preserving them in a near-native, hydrated state and avoiding artifacts caused by staining or dehydration. As amyloid fibrils form long, repeating structures, they can be imaged in 2D projections. The regularity of the amyloid fibrils, often supported with a helical symmetry, aids alignment and 3D reconstruction [133]. The resulting 3D density map reveals the stacking of β-strands, possible interactions between several protofilaments, and potential nucleic acid (NA) binding sites. Recent cryo-EM advances have offered unprecedented structural insights into various amyloid:nucleic acid complexes [75,134]. Nonetheless, deviations from perfect symmetry, filament curvature, or extreme flexibility can hinder helical reconstruction, limiting resolution to ~6–10 Å—as observed in recent studies of Hfq:DNA complexes [75].

Complementing high-resolution techniques, several faster, lower-resolution biophysical methods are used to characterize amyloids. A variety of microscopy techniques are applied. Besides transmission electron microscopy (TEM), fluorescence microscopy and atomic force microscopy (AFM) are commonly used. The dye Thioflavin T (ThT) is widely utilized due to its red-shifted fluorescence upon binding β-sheet aggregates [135], although its binding is not fully specific and can inhibit fibrillation in some contexts [136]. Super-resolution techniques such as stochastic optical reconstruction microscopy (STORM) and photoactivated localization microscopy (PALM) support kinetic studies of fibril growth and can reveal the polarity of assembly [137]. AFM provides nanometer-resolution topographic images, although its lateral resolution is lower than that of TEM [138]. It can operate in both air and liquid environments [139]. For amyloid imaging, fibrils are typically adsorbed onto flat surfaces like mica or lipid bilayers [75,140]. Recent advances have integrated AFM with infrared spectroscopy to map amyloid secondary structures at nanoscale resolution [141,142]. This may be used to map amyloid:NA complexes at the single fiber level in order to identify NA structural changes [143,144,145].

Spectroscopic analysis is indeed essential for probing secondary and super-secondary structures found in amyloids and their complexes. Fourier-transform infrared (FTIR) spectroscopy is widely used to analyze protein secondary structures through peptide bond vibrations. α-helices produce absorption peaks near 1655 cm^−1^, while β-sheets display characteristic peaks around 1630 and 1675 cm^−1^ [146,147]. Amyloid β-sheets, due to stronger hydrogen bonding, often exhibit peaks below 1625 cm^−1^ [148]. These spectra can be interpreted through peak fitting techniques. FTIR spectroscopy combined with hydrogen/deuterium exchange is particularly useful to predict super-secondary structures [149,150,151]. In parallel, NA structural change in the complex (sugar pucker, base pairing, etc.) can be analyzed using FTIR [145].

Synchrotron radiation circular dichroism (SRCD) enhances traditional CD spectroscopy by extending the spectral range and improving signal-to-noise ratio, allowing better analysis of β-rich and disordered proteins [152]. Amyloid fibrils are typically analyzed directly using SRCD [153]. BestSel, a modern algorithm, interprets CD spectra with high precision, differentiating between types of β-sheets [154]. Amyloids generally show a negative CD peak around 220 nm, especially when the unfolded protein signal appears near 200 nm [67]. Note that orientation or anisotropy with a longer-range scale in large amyloid fibril structures may cause CD spectrum distortion [155]. In such cases, the CD cell is mounted in a dedicated rotation chamber that allows automated rotation and averaging of spectra at various angles [156]. Nucleic acids complexed to amyloids can be analyzed using CD. Each NA conformation has a unique CD signature in the UV region (170–300 nm), and the effect of amyloid on this structure can be monitored using SRCD [67,126,157].

Electron paramagnetic resonance (EPR) spectroscopy offers further insights into side-chain dynamics, solvent accessibility, and secondary structures of amyloids [158]. EPR relies on the interaction between unpaired electrons and an external magnetic field, along with microwave radiation absorption. EPR studies of amyloids involve attaching paramagnetic tags to specific sites on the fibrils. The resulting spectra reflect the local structure and can be used to study flexibility and rigidity, as well as interactions with other macromolecules like proteins or nucleic acids. Spin mobility profiles can also indicate structural periodicities.

Small-angle scattering techniques also offer unique insights into the structural properties of amyloid assemblies, providing information on their size and shape at sub-nanometer to nanometer scales [159]. Among these, small-angle X-ray scattering (SAXS) is widely used for studying the overall structure of mainly single-component systems, offering valuable low-resolution models of amyloid assemblies in near-native conditions [160]. SAXS is particularly effective for analyzing single-component systems (amyloid fibrils) and can even provide models of protein–DNA complexes to some extent because of the difference in electron densities of protein and DNA molecules [161]. Small-angle neutron scattering (SANS), alternatively, excels at resolving specific components within multicomponent assemblies, such as amyloid–DNA complexes, through contrast variation using hydrogen–deuterium exchange [162]. Additionally, time-resolved SAXS and SANS have enabled the study of amyloid fibrillation kinetics [163,164]. Finally, X-ray fiber diffraction (complementary to SAXS) captures larger-scale fibril organization by detecting X-ray diffraction from one- or two-dimensionally ordered structures [165]. For example, this is useful for the analysis of a time course of structural changes of proteins in skeletal muscles during force development [166,167]. In amyloids, peaks near 0.7 and 1.35 Å^−1^ reflect inter-sheet (~8–10 Å) and inter-strand (~4.7 Å) distances (d = 2π/q). A particular emphasis must be placed on how differences in sample ordering impact the type and resolution of structural data obtained.

Inelastic neutron scattering (INS), using a neutron beam and complementary to FTIR spectroscopy [168], is used to investigate molecular vibrations and evaluate the conformational flexibility of amyloid fibers [169]. Measurements are typically conducted at 10 K in D_2_O ice using spectrometers like TOSCA at the ISIS Neutron and Muon Source (UK) [170]. TOSCA, an indirect time-of-flight spectrometer, spans the entire vibrational frequency range. Owing to the high incoherent scattering cross-section of hydrogen [171], the INS signal is predominantly influenced by hydrogen atom vibrations, which reflect self-correlated atomic motions [172]. This behavior can be quantitatively described using the Debye–Waller factor, which links structural rigidity to reductions in mean-square displacement. Consequently, INS provides direct insight into conformational changes in amyloid:DNA complexes, particularly those involving large-amplitude motions [75,173]. In addition to INS, elastic incoherent neutron scattering (EINS) and quasi-elastic neutron scattering (QENS) are useful to investigate diffusive molecular dynamics at the sub-nanosecond timescale and sub-nanometer length scale. Recent application of these techniques to amyloid fibrils suggests a correlation between the atom mobility and cytotoxicity [174,175].

## 7. Computational Approaches to Study Interactions Between Bacterial Amyloids and Nucleic Acids

In recent years, advances in artificial intelligence (AI)-based methods have revolutionized protein structure prediction. Some of these methods can predict the structure of a protein interacting with partners, including NA molecules. These computational approaches are powerful tools that support and guide experimental studies. However, although they are particularly well-suited for predicting the structure of globular proteins because their training set is essentially composed of this type of protein, their performance can be lower for amyloid peptides and even lower for their interaction with NA. Moreover, some of these approaches have inherent limitations that are not adapted to the specific study of amyloid:NA complexes, for example, the number of monomers, the size of the proteins, or the presence of NA molecules. Table 1 provides a selection of AI-based programs—AlphaFold 3 [176], RoseTTAFold2NA [177], Chai-1 [178], Boltz-1 [179], and Protenix [180]—that can predict the structures of proteins interacting with NA. These programs have all demonstrated their ability to correctly predict the structure of proteins, either alone or in the form of homomers or heteromers, whether they interact with DNA or RNA molecules or not. To the best of our knowledge, no study has yet evaluated the capability of these programs to predict the structures of complexes formed between amyloid peptides and nucleic acids.

To predict the structure of the amyloid:NA complex, it is first necessary to predict the structure of amyloid alone, which is the main challenging task [181]. Few studies have evaluated the ability of AI-based structure prediction programs to specifically predict amyloid structures. Thus, AlphaFold 2 [182] appears to have limitations in predicting cross-β amyloid conformations in amyloid peptides with antimicrobial properties [183]. Recently, Wojciechowska and coworkers [184] evaluated the structure prediction of six amyloid peptides using AlphaFold 2 and AlphaFold 3. As expected, AlphaFold 3 performs better than AlphaFold 2 on these examples. This difference is likely due to the reduced importance of multiple sequence alignments (MSAs) in AlphaFold 3. Moreover, interestingly, AlphaFold 3 can generate structures exhibiting the characteristic cross-β pattern observed in amyloids.

**Figure 2:**
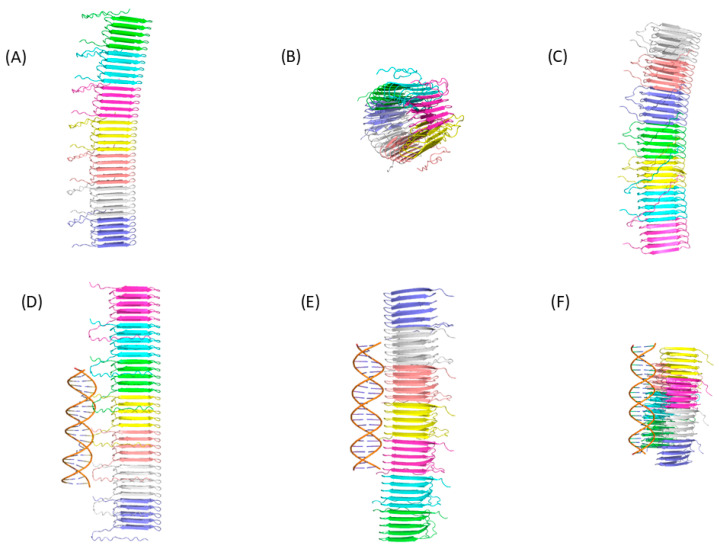
Structural predictions using webservers of three distinct IA-based programs. The structure of seven CsgA monomers was generated using (**A**) AlphaFold 3, (**B**) Chai-1, and (**C**) Protenix. Each monomer is presented with a different color. The structure of seven CsgA monomers in interaction with two monomers of DNA was generated using (**D**) AlphaFold 3, (**E**) Chai-1, and (**F**) Protenix. The DNA sequence used was a non-methylated CpG-containing duplex [118], namely GCCAACGGTGGCGCCAACGGTGGC (PDB ID 3QMG).

To illustrate the difficulty that current AI-based programs have in predicting the structure of amyloid peptide aggregation and their interaction with nucleic acids, we evaluated the ability of three web servers to predict the structure of seven monomers of the functional bacterial amyloid peptide CsgA from *E. coli* (Figure 2). We also evaluated their ability to predict the structure of seven CsgA monomers with DNA. To evaluate the quality of the structures generated, we compare the structural prediction of the seven CsgA monomers with the cryo-electron microscopy structure (PDB ID: 8ENQ) using the TM-score value [185]. A TM-score between 0.5 and 1.0 means that the protein has the same fold. The average TM-score for structures generated by AlphaFold 3 is 0.99, indicating AlphaFold 3’s success in predicting the correct structure of the 7 CsgA monomers, with or without DNA (see also [110]). On the contrary, Chai-1 and Protenix predict the structure of the seven CsgA monomers, with or without DNA, with an average TM-score of 0.4, indicating substantial deviations in peptide orientation or conformation relative to the experimental structure. These results suggest that, in this case study, these AI-based programs are not suitable for accurately predicting the correct structure. Interestingly, the DNA is correctly predicted as a double helix by the three programs (Figure 2). For Chai-1 and Protenix, the inclusion of the DNA double helix substantially modifies the global predicted arrangement of the seven CsgA monomers. However, the presence of the DNA double helix does not allow for structural predictions to be substantially closer to the experimental structure.

Several computational approaches have been developed specifically to predict the structure of amyloids. Among these, PACT (prediction of amyloid cross-interaction by threading) is a notable method designed to identify potential interactions between amyloidogenic peptides [186]. PACT is capable of predicting whether or not peptides with identical sequences are likely to aggregate, as well as assessing the potential for interaction between peptides with different sequences. PACT models amyloid structures by threading peptide sequences onto an amyloid fibril template. PACT can be accessed via a web server (https://pact.e-science.pl/pact/, accessed on 7 June 2025) for which the peptide sequences must contain between 14 and 45 amino acids. If a peptide sequence exceeds 45 residues, the user can use the standalone version available at the GitHub repository: https://github.com/KubaWojciechowski/PACT, accessed on 7 June 2025). The two main limitations of PACT are the inability to select the number of monomers and the impossibility of considering the presence of NA molecules. PACT has been applied to investigate interactions between CsgA and human disease-related peptides such as hIAPP, a protein involved in type 2 diabetes or the alpha-synuclein, a protein linked to Parkinson’s disease. Notably, PACT successfully predicted specific CsgA fragments (R1 and R5) capable of interacting with hIAPP and identified the key interacting region of alpha-synuclein (residues 32–56) in contact with various bacterial CsgA variants. These predictions were supported by experimental validation [186].

One of the most recent developments in amyloid structure prediction is RibbonFold, a new AI-driven tool that is capable of predicting the structure of amyloid aggregation [187]. Unlike most of the other AI-based tools designed to predict protein structures, RibbonFold incorporates amyloid-specific constraints in its prediction algorithm and addresses the fact that amyloids are capable of adopting multiple stable polymorphic forms, depending not only on the sequence but also on a variety of conditions, such as environment, mutations, etc. RibbonFold has been adapted from AlphaFold 2. It incorporates several key modifications, such as parallel in-register β-sheet constraints and a polymorph-aware loss function in order to model the structural polymorphism of amyloid fibrils. Moreover, it uses random MSA subclustering to enhance structural diversity. RibbonFold was applied to peptides that are known to exist in amyloid form, such as Aβ, tau, and α-synuclein [187]. It successfully recovered known polymorphs and identified novel ones. RibbonFold significantly outperformed AlphaFold 2 and AlphaFold 3. Although RibbonFold has not yet been extensively used to model amyloid:NA complexes, its ability to explore multiple stable structures suggests that it would outperform the current available computational tools.

## 8. Conclusions

**Figure 3:**
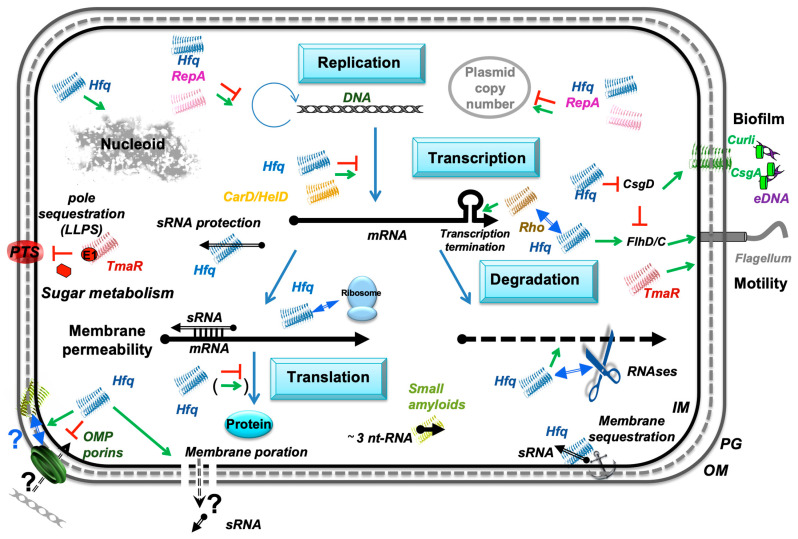
Amyloid-Driven Nucleic Acid Interaction Network in Bacteria. Recent discoveries have highlighted the significant roles bacterial amyloids play through their interactions with nucleic acids. These interactions affect both cellular regulation and structural organization. RepA and Hfq amyloid proteins control replication/plasmid copy number and DNA can induce amyloidogenicity. Similarly, extracellular DNA (eDNA) has been implicated in promoting amyloid formation in bacterial biofilms, particularly with curli fibers in pathogenic bacteria. Conversely, amyloids influence curli expression and biofilm formation. Bacterial amyloids also interact with RNA, as demonstrated with proteins like Rho, CarD, HelD, TmaR, and Hfq, impacting gene expression. Hfq indeed exemplifies the dual nucleic acid-binding capacity of bacterial amyloids, influencing both RNA and DNA structure and function via its C-terminal amyloid domain. Collectively, these findings underscore the multifaceted regulatory and structural roles of amyloid-like assemblies in bacterial cells. The RNAs are shown as black arrows and DNA as grey double helices; 5’ and 3’ ends of the mRNA are depicted by a “ball and arrowhead”, respectively; positive and negative regulations are indicated by green arrows and red T-shape symbols, respectively; double blue arrow indicates a physical interaction with another protein; dotted line symbolizes peptidoglycan (PG) between outer (OM) and inner (IM) membranes.

Bacterial cells can produce a variety of proteins that form amyloid-like structures. Contrary to amyloids occurring in humans and animals, those of bacterial origin often play important physiological roles. Importantly, some of the functions of these bacterial amyloids are dependent on their interactions with nucleic acids. These include the formation of biofilms with extracellular curli–DNA complexes as major constituents of these structures, regulation of replication of extrachromosomal DNA elements (like plasmids and bacteriophage genomes), and genetic recombination by DNA-interacting proteins through their amyloid domains (like WH1 of RepA and C-terminal domain of Hfq), as well as the control of gene expression by modulating interactions between small RNAs and their target mRNAs (e.g., by the C-terminal domain of Hfq) (Figure 3). In addition to their roles in bacterial cell physiology, DNA-bacterial amyloid complexes can contribute to the development of neurodegenerative and inflammatory diseases, through the stimulation of pathological human/animal amyloid formation and induction of auto-inflammation processes, respectively.

## Figures and Tables

**Table 1:** AI-based program available for the structure prediction of protein:NA complexes.

Webserver Name.	Address (accessed on 7 June 2025)	User Quota (Jobs/Day)	Size Limit (Residue)	Code (accessed on 7 June 2025)
AlphaFold 3	https://alphafoldserver.com/	30	5120	https://github.com/google-deepmind/alphafold3
RoseTTAFold2NA	Not Available			https://github.com/uw-ipd/RoseTTAFold2NA
Chai-1	https://lab.chaidiscovery.com/	25	2048	https://github.com/chaidiscovery/chai-lab
Boltz-1	Not Available			https://github.com/jwohlwend/boltz
Protenix	https://protenix-server.com/add-prediction	No quota	2560	https://github.com/bytedance/Protenix

## Abbreviations

The following abbreviations are used in this manuscript: NA: nucleic acid; sRNA: small noncoding RNA; eDNA: extracellular DNA; IM/OM: inner/outer membrane; LLPS: liquid–liquid phase separation; ssNMR: solid-state nuclear magnetic resonance spectroscopy; SRCD: synchrotron radiation circular dichroism; FTIR: Fourier transform infrared spectroscopy; EPR: electron paramagnetic resonance; (T)EM: (transmission) electron microscopy; AFM: atomic force microscopy (AFM); SAXS/SANS: small angle X-ray or neutron scattering; INS: inelastic neutron scattering; EINS: elastic incoherent neutron scattering: QENS: quasi-elastic neutron scattering; IDP/IDR: intrinsically disordered proteins or regions.
